# Supplementary material for: Chromothripsis during telomere crisis is independent of NHEJ, and consistent with a replicative origin
Source: Genome Res. 2019 May;29(5):737–49. doi: 10.1101/gr.240705.118 (PMC6499312; doi:10.1101/gr.240705.118)
Supplement: Supplemental Material [file supp_gr.240705.118_Supplemental_file_1.zip › contigs/annotated_contigs/DB53/contig.2.DB53_length_374_mean_cov_25.1844919786.docx]

**DB53_length_374_mean_cov_25.1844919786**

```
AACTAGCCTCGCCAACACGGCGAAACCCTGTCTCTACTAAAAATACAAAAGTTAGCTGGGCGTGGTGGCGTGTGCCTGTAATCCCAGCT
 >chr7:152371496-152371790 + E=1e-135 p=1e-03
ACTCGGGAGGCTGAGGCAGGAGAATCGCTTGAACCCGGGAGGTGGAGGTTGCAGTGAGCCGAGATCACACCATCGCACTCCAGCCTGGG

CGACAGAGCGAGAGTTCATCTCAAAAAAATATATATTTATATATAAAAAATATGTTTATATGTATTATATACTTTCTATATAATCTATA

TTATAAATATATTTTTAAATATATATT|ATATATAAAAATATA|TTTGTATTGTTAGTAGAGACGGCATTTCTCCATGTGGGTCAGGCT
                                           >chrUn_gl000220:143168-143231 + E=2e-26
GGTCTCGAACTGGCGACC|TC
```
